# Smoking and cancer: Brazil and the Global Burden of Disease initiative: Fumo e câncer: Brasil e a iniciativa "Carga Global de Doenças"

**DOI:** 10.1590/1516-3180.2015.13353108

**Published:** 2015-08-21

**Authors:** Paulo Andrade Lotufo

**Affiliations:** I MD, DrPH. Full Professor, Department of Internal Medicine, Faculdade de Medicina da Universidade de São Paulo (FMUSP), São Paulo, Brazil.

The Global Burden of Disease (GBD) study is an initiative from the World Health Organization and Harvard University. It was launched in 1996 as an initiative by Alan Lopez and Christopher Murray. The aim was to provide systematic epidemiological estimates for an unprecedented 150 major health conditions and, from this, indispensable global and regional data for health planning, research and education.^1^ Subsequently, with financial support from the Bill and Melinda Gates Foundation, the University of Washington embraced the proposal for an Institute of Health Metrics. Since then, the GBD initiative has sought to "synthesize all available epidemiological data using a coherent measurement framework, standardized estimation methods and transparent data sources to facilitate comparisons of health loss over time across causes, age-sex groups and countries."^2^

The GBD initiative has been developed along two important lines of action. One is to involve a considerable number of researchers around the world, in order to establish a continuous collaboration with interchangeability of information. The other summarizes measurements such as disability-adjusted life years (DALYs) and healthy life expectancy (HALE), in order to enable comparison of epidemiological variables over time and across countries. A further line of action, which is to be launched in 2016-2017, will describe epidemiological patterns according to states/provinces/departments in the US, Mexico, Kenya, China, India and Brazil (personal information).

This Editorial provides a brief overview of the smoking epidemic in Brazil, in comparison with other countries that have relevance to our country, either due to the geopolitical context or because of geographical and historical ties. Thus, we chose the following for comparison purposes: Argentina, China, Germany, India, Italy, Japan, Mexico, Portugal, Russia, South Africa, Spain, the United Kingdom (UK) and the United States of America (USA). We restricted the comparison only to DALY information forfor traditional cancer types that are linked directly to smoking exposure.

Table 1 shows results published previously by the GBD team regarding smoking prevalence.^3^ The prevalence rate for the smoking habit in 2010 was lower in Brazil than the rates in those countries, with the exception of Mexico and India. The decline of smoking prevalence rates from 1980 to 2010 was faster in Brazil (-34%) than the average decline among the other countries (-28%). The smoking data for Brazil was derived from VIGITEL, an annual survey by means of telephone calls in the 27 state capitals, in which the overall prevalence matched that of the Brazilian National Health Survey.^4^

**Table 1:**
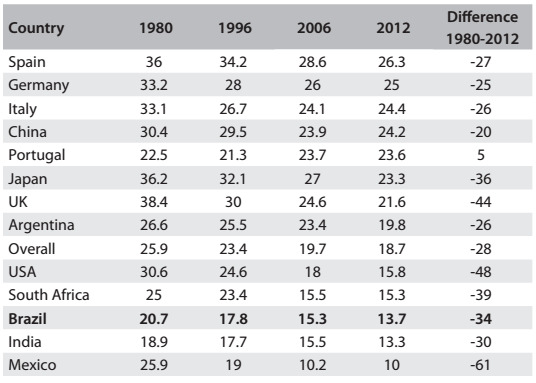
Temporal trends of prevalence rates for the smoking habit (percentage) in selected countries, according to the compilation of the Global Burden of Diseases initiative. Reproduced from Ng et al.^3^

**Table 2:**
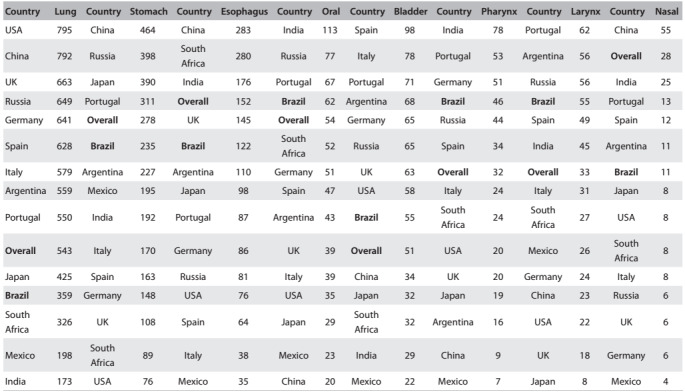
DALY (disability-adjusted life years) values (x 1000), according to selected countries and tobacco-related cancer sites.

Source: Global Burden of Disease Cancer Collaboration^5^

Table 2 shows the DALYs for cancer for both sexes, according to estimates from the GBD team published recently.^5^ The cancers analyzed related to:

1. lung, trachea and bronchus;
2. stomach;
3. esophagus;
4. lip and oral cavity;
5. pharynx;
6. larynx; and
7. urinary bladder.

The following initial observations can be made:

1. #1: Although lung cancer is the leading cause of cancer mortality in Brazil, accounting for 12% of the almost 200,000 cancer deaths in 2013,^6^ comparison with these other countries revealed that the frequency of this type of malignant neoplasm among Brazilians was lower.
2. #2: The DALY values due to upper aerodigestive cancers (nasal, oral, pharyngeal, laryngeal and esophageal) were relatively higher in Brazil than in these other countries. For oral cancer, Brazil was ranked third.
3. #3: Despite the decline in gastric cancer mortality rates worldwide and in Brazil, this type of malignant neoplasm had higher frequency in Brazil than in other Western countries.
4. #4: The DALY values in Portugal were closer to those reported in Brazil for stomach, nasal, oral, laryngeal and pharyngeal cancers.
5. #5: The highest DALYs for nasal, oral, pharyngeal, laryngeal and esophageal cancers were in India, the country with the lowest smoking prevalence, followed by South Africa, China and Brazil.
6. #6: Argentina had DALY values closer to Brazil for stomach, esophageal and laryngeal cancer.

We are not discussing the causes and effects of risk factors for smoking-related cancers at this moment. We are only calling for public policy action. Successful action to control smoking in Brazil will certainly not contain the impact of other smoking-related cancers such as those located in the trachea, bronchus and lung, or the impact of upper aerodigestive malignant neoplasms.
